# CD226^+^ B cells in primary Sjögren’s syndrome: a key player in clinical manifestations and disease pathogenesis

**DOI:** 10.3389/fimmu.2025.1623774

**Published:** 2025-07-25

**Authors:** Ping Zhao, Saizhe Song, Song Zhang, Cheng Peng, Wei Cheng, Xin Chang, Changhao Xie, Zhongli Hu, Cuiping Liu

**Affiliations:** ^1^ Jiangsu Institute of Clinical Immunology and Jiangsu Key Laboratory of Clinical Immunology, The First Affiliated Hospital of Soochow University, Suzhou, China; ^2^ Department of Rheumatology and Clinical Immunology, The First Affiliated Hospital of Bengbu Medical University, Bengbu, China; ^3^ Department of Rheumatology, The First Affiliated Hospital of Soochow University, Suzhou, China; ^4^ Department of Dermatology, Changshu No 2 People’s Hospital, Suzhou, China; ^5^ Department of Hematology, The First Affiliated Hospital of Bengbu Medical University, Bengbu, China

**Keywords:** CD226, costimulatory molecule, B cells, cytokines, primary Sjögren’s syndrome

## Abstract

**Introduction:**

Primary Sjögren’s syndrome (pSS) is a systemic autoimmune disorder characterized by lymphocytic infiltration of exocrine glands, leading to sicca symptoms and systemic complications. CD226, a co-stimulatory receptor implicated in the pathogenesis of multiple autoimmune diseases including systemic lupus erythematosus (SLE), rheumatoid arthritis(RA), and pSS, regulates immune cell activation. However, the specific role of CD226+ B cells in pSS pathogenesis remains unclear. This study aims to elucidate the functional contribution of CD226^+^ B cells to pSS development and their clinical relevance.

**Methods:**

The percentages of CD226 on T cells, B cells, CD56^+^ NK cells and CD14^+^ monocytes in the peripheral blood(PB) of pSS patients and healthy controls (HCs) were detected by flow cytometry.Multicolor flow cytometry was employed to examine the distribution of CD226 in B cell subsets of pSS patients, as well as the expression levels of co-stimulatory molecules, activation and proliferation markers, immunoglobulins, and pro-inflammatory cytokines on both CD226^+^ B cells and CD226^-^ B cells. Multicolor immunofluorescence staining was applied to detect the co-expression of B cells and CD226 in the salivary gland of pSS patients.Microarray analysis was conducted to analyze the transcriptomic profiles of sorted CD226^+^ CD19^+^ B cells and CD226^-^ CD19^+^ B cells.

**Results:**

CD226 expression in the peripheral blood of pSS patients was significantly increased on T cells, CD19^+^ B cells and CD14^+^ monocytes, but significantly decreased on CD56^+^ NK cells.We identified a distinct CD226^+^CD19^+^ B cell subset that exhibited pathogenic features in pSS. CD226 was significantly upregulated on B cells in the peripheral blood and salivary glands of pSS patients.CD226^+^ CD19^+^ B cell showed a stronger correlation with clinical features, disease activity, and prognosis in pSS patients.The ROC curve demonstrated that CD226^+^ CD19^+^ B cell exhibited significant diagnostic capability to distinguish pSS patients from healthy controls and to differentiate disease activity.This subset also exhibited heightened activation and pro-inflammatory phenotypes.

**Discussion:**

CD226^+^ B cells are expanded in pSS, strongly correlating with clinical manifestations and disease activity. These cells display enhanced effector profiles (activation, cytokine/immunoglobulin production) and demonstrate diagnostic utility. Our findings identify CD226^+^ B cell as a pathogenic driver in pSS, positioning CD226 as a promising novel therapeutic target and biomarker.

## 1 Introduction

Primary Sjögren’s syndrome (pSS) is a chronic systemic autoimmune disease characterized by T cell-mediated B cell hyperactivation and dysregulated cytokine production (1). Histopathologically, it is typically defined by lymphocytic infiltration of exocrine glands, primarily composed of B cells, T cells, and antigen-presenting cells (2). B lymphocyte hyperactivity plays a pivotal role in pSS pathogenesis, evidenced by the presence of autoantibodies (e.g., rheumatoid factor (RF), anti-SSA, and anti-SSB) and elevated serum polyclonal immunoglobulin levels. Moreover, B cells contribute to pSS progression via cytokine secretion and antigen presentation (3–5).

It has been established that the costimulatory signaling pathway plays a critical role in orchestrating T cell-dependent B cell hyperactivation in pSS. To date, two major families of costimulatory molecules—the immunoglobulin superfamily (IgSF) and the tumor necrosis factor superfamily(TNFSF)—have been identified to mediate the interactions among antigen-presenting cells, B cells, and T cells, thereby promoting pSS progression (6–10). CD226, a type I transmembrane glycoprotein, belongs to IgSF, which is broadly expressed in various immune cells, including peripheral blood monocytes, T cells, natural killer (NK) cells, B cells and dendritic cells (11). The interaction between CD226 and its ligands is essential in mediating diverse immune responses, including promoting T cell activation and proliferation, enhancing NK cell cytotoxicity, strengthening intercellular adhesion, and modulating lymphocyte signal transduction and cytokine secretion (11, 12).

It was found that CD226 was expressed in subsets of B cells, such as memory B cells, plasmablasts and plasmacytes in human peripheral blood, and was up-regulated upon stimulation with CpG-ODN. In addition, after stimulation with CpG-ODN, CD226 was involved in the production of IL-10 and antibody by B cells, indicating that CD226 participated in B cell-mediated immune responses (13). Our previous research demonstrated that CD226 expression on CD14^+^ monocytes of peripheral blood from patients with pSS was elevated and exhibited a positive correlation with disease activity (14). Furthermore, we also found that plasma sCD226 was significantly correlated with the clinical features of pSS, and can be regarded as a biomarker of pSS disease activity (15). Base on the basis of the above research background, This study sought to examine the manifestation and clinical relevance of CD226 on B cells in subjects with pSS, and to explore the immunologic function by which CD226 mediated B cell in the context of pSS pathology.

## 2 Materials and methods

### 2.1 Patients

In our study, 40 pSS patients and 26 healthy controls (HCs) were recruited as research subjects. All pSS patients were diagnosed according to the classification criteria of Sjögren’s syndrome revised by American-European consensus group (AECG) in 2002 (16), and none of them received medication. We exclusively enrolled treatment-naïve patients at initial diagnosis, with documented absence of previous immunomodulatory therapy (including systemic corticosteroids, cytokine inhibitors, or other immunosuppressants). Clinical data and laboratory parameters of pSS patients were collected. Based on the ESSDAI score (17), pSS patients with ESSDAI≥5 were classified as active group, while those with ESSDAI<5 were considered inactive group. Fresh venous peripheral blood samples were collected from pSS patients and HCs with fasting in the morning after an overnight fast. Partial patients underwent pathological examination of salivary gland(SG) biopsy, and the remaining SG tissue was preserved after inspection for our study. Our study was approved by the Ethics Committee of the First Affiliated Hospital of Soochow University in 2020 (Ethical No. 2020105).Informed consent was obtained from all study participants.

### 2.2 Mice

Non-obese diabetic (NOD) mice were utilized as mice models for pSS, and institute of cancer research(ICR) mice were taken as mice models for control mice (18, 19). The NOD(n=5) and ICR(n=5) mice(female, aged 6 weeks) were purchased from Changzhou Cavens Experimental Animal Co., Ltd. The animal research was approved by the Animal Ethics Committee of Soochow University(Ethics No. SUDA20240724A03).

### 2.3 Antibodies

Fluorescence-conjugated anti-human monoclonal antibodies were adopted in this study as following: anti-CD3-FITC, anti-CD8-PE-Cy5, anti-CD19-PE-Cy5, anti-CD56-FITC, anti-CD14-FITC, anti-CD226-PE, anti-CD27-PE-Cy7, anti-IgD-FITC, anti-CD21-APC-Cy7, anti-CD38-AF700, anti-CD80-PE-Cy7, anti-CD86-FITC, anti-CD137-APC-Cy7, anti-CD69-AF700, anti-IgG-PE-Cy7, anti-IgA-FITC, anti-Ki67-AF700, anti-IFN-γ-PE-Cy7, anti-TNF-α-PE-Cy7. And fluorescence-conjugated anti-mouse monoclonal antibodies were utilized as following: anti-CD3-PE-Cy5, anti-CD4-FITC, anti-CD8-PE-Cy7, anti-NK1.1-APC-Cy7, anti-B220-FITC, anti-CD11b-PerCP-Cy5.5, anti-CD226-PE. These above anti-human and anti-mouse monoclonal antibodies were all purchased from Biolegend (USA).

### 2.4 Flow cytometry

The human anti-CD3, anti-CD8, anti-CD19, anti-CD56, anti-CD14, anti-CD27, anti-IgD, anti-CD21, anti-CD38, anti-CD80, anti-CD86, anti-CD137, anti-CD69, anti-IgG, anti-IgA, and anti-CD226 antibodies were added into 50µL peripheral blood samples and incubated for 30 minutes. Then erythrocyte lysis was added into the samples for red blood cell lysis. For intracellular staining, peripheral blood mononuclear cells(PBMCs) were isolated from human samples, and added phorbol 12‐myristate 13‐acetate (PMA) and ionomycin for stimulating cells, then stained by human anti-CD19 and anti-CD226 antibodies. Subsequently, Fixation and permeabilization buffers were added, followed by staining with human anti-Ki67, anti-IFN-γ, and anti-TNF-α antibodies. All samples were conducted by flow cytometry. For NOD and ICR mice, spleen single-cell suspensions were prepared and stained with mouse anti-CD3, anti-CD4, anti-CD8, anti-NK 1.1, anti-B220, anti-CD11b and anti-CD226 antibodies for processing by fow cytometry(Cyto FLEX, Beckman Coulter, USA).All the data were analyzed by FlowJo 10.8.1.

### 2.5 Immunohistochemistry and immunofluorescence staining

Paraffin sections of SG tissues from pSS patients and HCs were deparaffinized, underwent antigen retrieval, and were blocked. Subsequently, the sections were incubated with a human anti-CD226 antibody as the primary antibody, followed by incubation with a goat anti-mouse IgG antibody as the secondary antibody. Then 3,3’-Diaminobenzidine(DAB) staining was utilized to detect the expression of CD226 in SGs. Using tyramide signal amplification(TSA) fluorescence labeling technique, the co-expression of CD226 and CD20 in SGs tissue was detected according to the aforementioned method, utilizing human anti-CD226 and human anti-CD20 antibody respectively. The nucleus was stained by 4’,6-diamidino-2-phenylindole(DAPI), CD226 was labeled by red fluorescence, and CD20 was labeled by green fluorescence.

### 2.6 Microarray analysis

Using a fluorescence-activated cell sorter (FACS) to sort CD226^+^ CD19^+^ B cells and CD226^−^ CD19^+^ B cells from splenocytes of NOD mice. Total RNA was extracted with Trizol (Invitrogen) and assessed with Agilent 2100 BioAnalyzer(Agilent Technologies, Santa Clara, CA, USA) and Qubit Fluorometer (Invitrogen). RNA-seq libraries were generated and sequenced by CapitalBio Technology (Beijing, China). The cDNA was synthesized from the extracted RNA using a reverse transcription kit, and labeled with fluorescent dyes. The purified DNA was denatured at high temperature (typically at 95°C for 3 minutes), followed by hybridization of the labeled cDNA to the mouse gene expression microarray chip(Agilent) according to the manufacturer’s instructions. The hybridization was then carried out in a hybridization oven at 42°C for 16–20 hours. The microarray chip was washed to remove unbound cDNA using the recommended buffers and then scanned to detect fluorescence signals.

The gene expression analyses were performed with StringTie(v1.3.3b). DESeq(v1.28.0) was used to analyze the differentially expressed genes(DEGs) between samples. Thousands of independent statistical hypothesis testing was conducted on DEGs, separately. Then a p-value was obtained, which was corrected by FDR method. The corrected P-value (q-value) was calculated by correcting using BH method and used to conduct significance analysis. Parameters for classifying significantly DEGs were≥2-fold differences (|log2FC|≥1, FC: the fold change of expressions) in the transcript abundance and p ≤ 0.05. The annotation of the DEGs were performed based on the information obtained from the database of ENSEMBL, NCBI, Uniprot, GO, and Kyoto encyclopedia of genes and genomes(KEGG).

### 2.7 Statistical analysis

Flow cytometry data were processed and analyzed using FlowJo software (version 10.8.1). Images were processed using ImageJ software. Statistical analysis and data visualization were performed using GraphPad Prism software (version 8.0.2). The Shapiro-Wilk test was applied to assess data normality (n<50). Normally distributed measurement data were expressed as mean ± standard deviation ( $\bar{\text{x}}$ ± s), while non-normally distributed data were presented as median (minimum, maximum). Comparisons between two groups were analyzed using the Student’s t-test for normally distributed data and the Mann-Whitney U test for non-normally distributed data. For correlation analysis, the Pearson correlation coefficient was used for normally distributed data, and the Spearman correlation coefficient was applied for non-normally distributed data. *P*-value<0.05 was considered statistically significant, with significance levels denoted as follows: *P<0.05, **P<0.01, and ***P<0.001, ****P<0.0001.

## 3 Results

### 3.1 The expression of CD226 on B cells increased in pSS patients

The pSS patients who participated in this study were matched with HCs by gender, and there was no statistical difference in age (46.75 ± 13.10 *vs*. 44.35 ± 9.28 (years), *P*>0.05).The basic information of pSS patients and HCs enrolled in this study is shown in **Table 1**.

**Table 1:** Basic information of the participants in this study.

Characteristics	pSS	HCs
number(n)	40	26
age(years)	46.75 ± 13.10	44.35 ± 9.28
male, n (%)	2(5%)	1(3.8%)
female, n (%)	38(95%)	25(96.2%)
disease duration (months)	42(1, 216)	NA
Major clinical features		
xerostomia, n (%)	36 (90%)	NA
xerophthalmia, n (%)	26 (72.5%)	NA
decayed tooth, n (%)	10 (25%)	NA
gland swelling, n (%)	5 (12.5%)	NA
Raynaud’s phenomenon, n (%)	3 (7.5%)	NA
fatigue, n (%)	29 (72.5%)	NA
weight reduction, n (%)	2 (5%)	NA
arthralgia, n (%)	15 (37.5%)	NA
skin involvement, n (%)	3 (7.5%)	NA
PBC, n (%)	6 (15%)	NA
renal injury, n (%)	5 (12.5%)	NA
ILD, n (%)	12 (30%)	NA
fever, n (%)	2 (5%)	NA
muscle involvement, n (%)	1 (2.5%)	NA
Major laboratory features		
ESR (mm/h)	14 (2, 70)	NA
CRP (mg/L)	2.14 (0.20, 22.79)	NA
RF (IU/mL)	30 (2.6, 777)	NA
WBC(×10^9^/L)	5.30(2.69, 11.78)	NA
lymphocyte (×10^9^/L)	1.57 ± 0.56	NA
neutrophil (×10^9^/L)	3.20 (1, 8.74)	NA
RBC (×10^12^/L)	4.33 ± 0.41	NA
Hb (g/L)	125.8 ± 12	NA
platelet (×10^9^/L)	205.2 ± 58.48	NA
serum globulin (g/L)	30.45 (21.8, 49.5)	NA
IgG (g/L)	16.45 (9.27, 31.8)	NA
IgA (g/L)	2.78 (1.52, 10.6)	NA
IgM (g/L)	1.20 (0.52, 7.35)	NA
C3 (g/L)	0.92 (0.62, 1.52)	NA
C4 (g/L)	0.20 (0.07, 0.44)	NA
Anti-Ro52 (+), n (%)	34 (85%)	NA
Anti-Ro60 (+), n (%)	32 (80%)	NA
Anti-SSB (+), n (%)	22 (55%)	NA
Anti-centromere (+), n (%)	5 (12.5%)	NA
ESSDAI	5 (1, 18)	NA
ESSPRI	3.92 ± 1.10	NA

ESR, Erythrocyte Sedimentation Rate; CRP, C-Reactive Protein; RF, Rheumatoid Factor; WBC, White Blood Cell; RBC, Red Blood cell; Hb, hemoglobin; IgG, Immunoglobulin G; IgA, Immunoglobulin A; IgM, Immunoglobulin M; C3, Complement 3; C4, Complement 4; NA, Not Applicable; PBC, primary biliary cholangitis; ILD, Interstitial Lung Disease; ESSDAI, European League Against Rheumatism Sjogren’s Syndrome Disease Activity Index; ESSPRI, European League Against Rheumatism Sjogren’s Syndrome Patient Reported Index.

**Figure 1:**
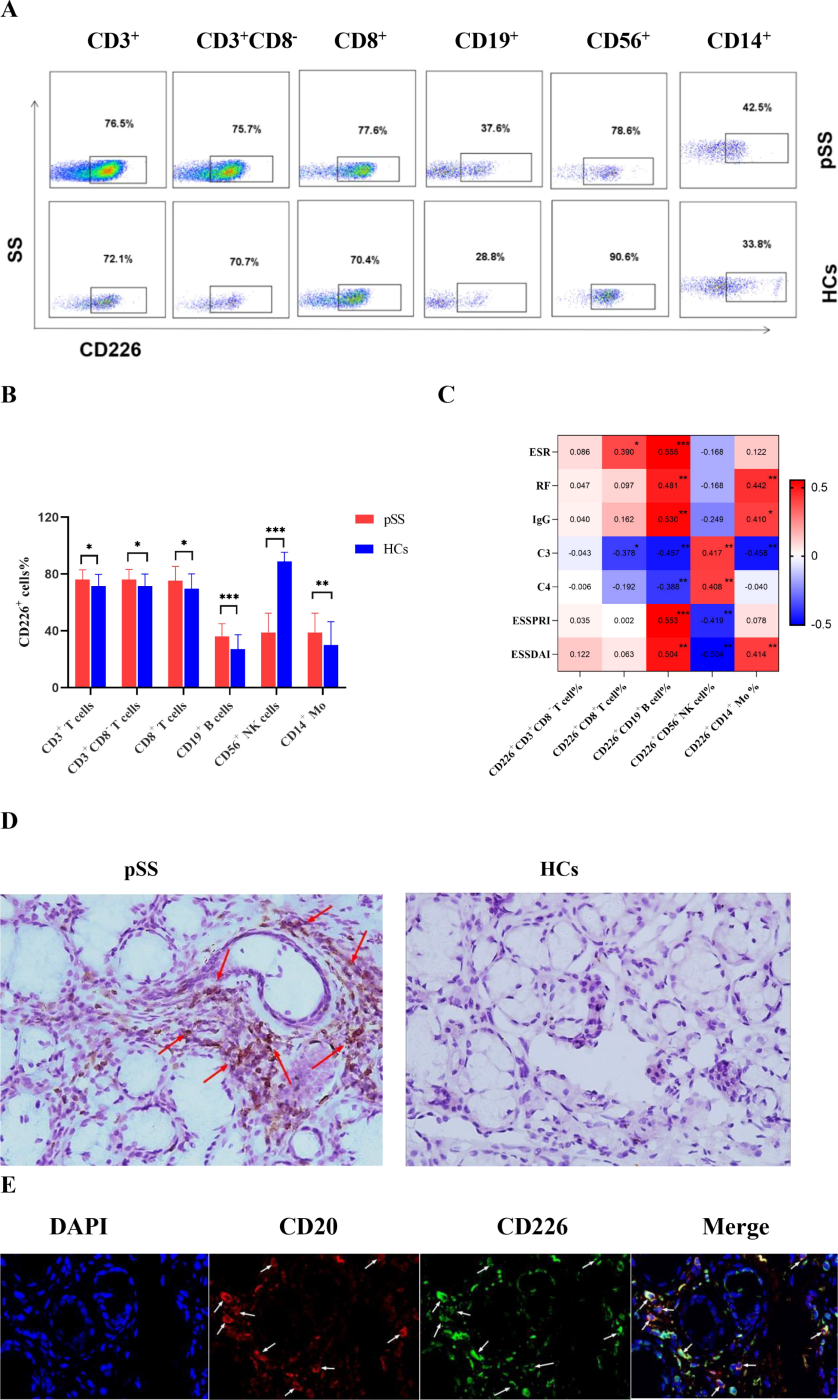
The expression of CD226 in pSS patients and HCs. **(A)** The representative FACS plots of CD226 on CD3^+^ T cells, CD3^+^CD8^-^ T cells, CD8^+^ T cells, CD19^+^ B cells, CD56^+^ NK cells and CD14^+^ monocytes in pSS patients(n=40) and HCs(n=26). **(B)** The percentage of CD226^+^CD3^+^ T cells, CD226^+^CD3^+^CD8^-^ T cells, CD226^+^CD8^+^ T cells, CD226^+^CD19^+^ B cells, CD226^+^CD56^+^ NK cells and CD226^+^CD14^+^ monocytes in peripheral blood of patients with pSS and HCs(Student’s t-test and Mann-Whitney U test). **(C)** Correlations between the expression of CD226 on T cells, CD19^+^ B cells, CD56^+^ NK cells,CD14^+^ monocytes and clinical parameters in pSS patients(Spearman’s rank correlation coefficient test,*P<0.05,**P<0.01,***P<0.001 ). **(D)** The expression of CD226 in salivary gland of patients with pSS and HCs by immunohistochemistry assay (20x). **(E)** The expression of CD226^+^CD20^+^ B cells in salivary gland of patients with pSS by immunofluorescence staining (blue for DAPI, red for CD20, green for CD226).

Flow cytometry was utilized to identify the expression levels of CD226 on T cells, B cells, CD56^+^ NK cells and CD14^+^ monocytes in the peripheral blood of pSS patients and HCs. The gating strategies are depicted in **Supplementary Figure 1**. The results revealed that, compared with HCs, the percentage of CD226 on CD3^+^ T cells, CD3^+^CD8^-^ T cells,CD8^+^ T cells as well as CD19^+^ B cells and CD14^+^ monocytes was significantly increased in patients with pSS(**Figure 1B**). In contrast, CD226 percentage on CD56^+^ NK cells was significantly reduced in pSS patients compared to HCs. Representative FACS plots are shown in **Figure 1A**. Next, we conducted a correlation analysis between the expression levels of CD226 on CD3^+^CD8^-^ T cells, CD8^+^ T cells, CD19^+^ B cells, CD56^+^ NK cells, and CD14^+^ monocytes and clinical activity parameters in patients with pSS. The findings indicated that in pSS patients, the percentage of CD226^+^CD3^+^CD8^-^ T cells did not exhibit any significant correlation with the clinical parameters of pSS. The percentage of CD226^+^ CD8^+^ T cells demonstrated a positive correlation with erythrocyte sedimentation rate (ESR), while it showed a negative correlation with complement C3 (C3). However, no significant correlation was observed between the percentage of CD226^+^ CD8^+^ T cells and rheumatoid factor (RF), immunoglobulin G (IgG), European League Against Rheumatism Sjögren’s Syndrome Patient-Reported Index (ESSPRI), European Sjögren’s Syndrome Disease Activity Index (ESSDAI), or complement C4 (C4). The percentage of CD226^+^CD56^+^ NK cells was positively correlated with C3 and C4, while negatively correlated with ESSPRI and ESSDAI, and not correlated with ESR, RF and IgG. There was a positive correlation between the percentage of CD226^+^CD14^+^ monocytes and RF, IgG and ESSDAI, and a negative correlation with C3, but no correlation with ESR, ESSPRI and C4. A significant positive correlation was exhibited between the percentage of CD226^+^CD19^+^ B cells and ESR, RF, IgG, ESSPRI and ESSDAI. Conversely, a significant negative correlation was identified between CD226^+^CD19^+^ B cells and C3 and C4 (**Figure 1C**). In brief, the findings demonstrated that CD226^+^CD19^+^ B cells were more closely related to the disease activity and clinical parameters of pSS patients. We further discovered that CD226 was expressed in the SG tissue of pSS patients and co-expressed with CD20^+^ B cells (**Figures 1D, E**).

### 3.2 The expression of CD226^+^ CD19^+^ B cells was related to the clinical features and disease activity of pSS patients

**Figure 2:**
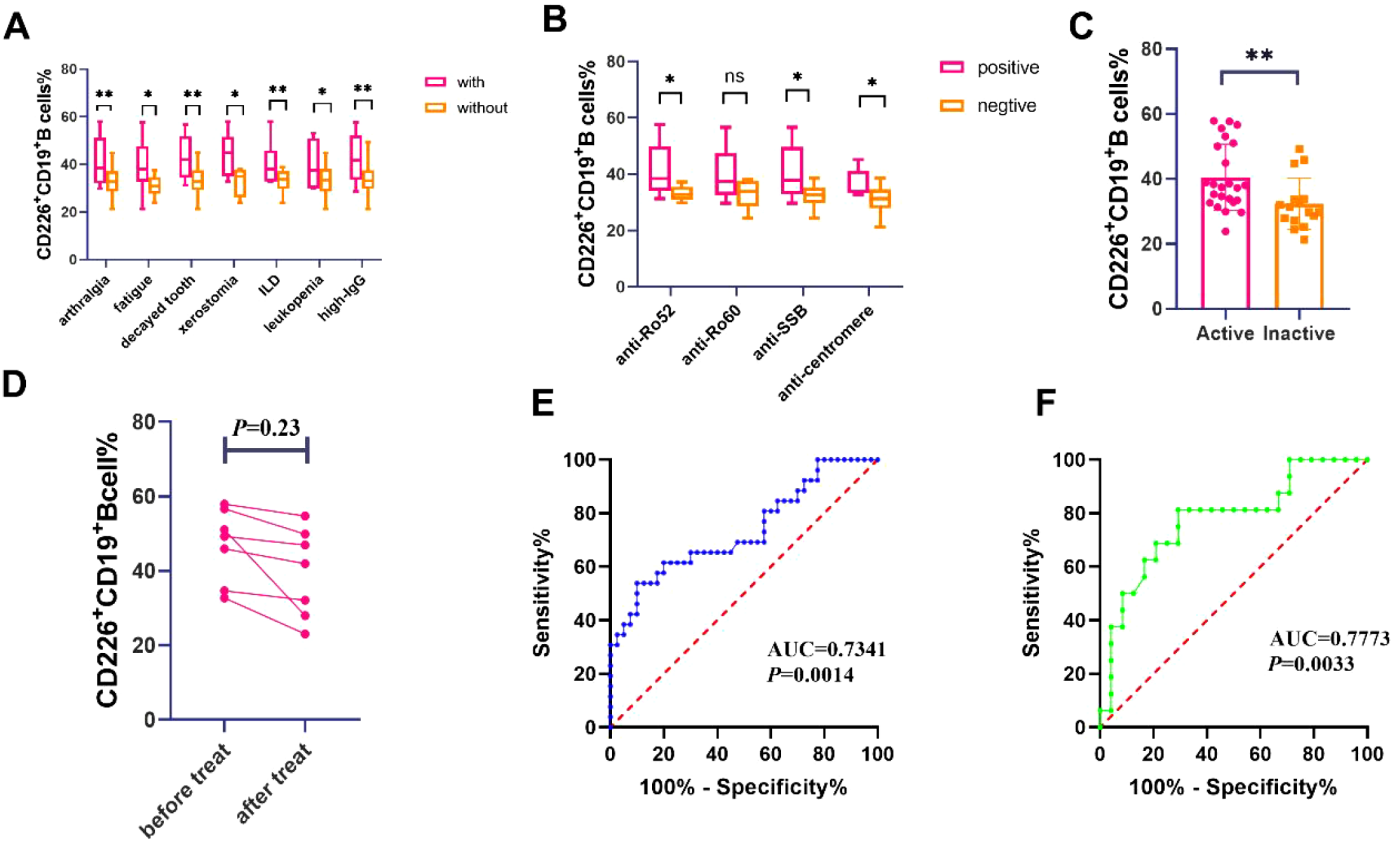
Correlations between CD226^+^CD19^+^B cells in peripheral blood and clinical features of patients with pSS(n=40). **(A)** Percentage of CD226^+^CD19^+^ B cells in pSS patients with different clinical manifestations by flow cytometry(Student’s t-test and Mann-Whitney U test) *P < 0.05; **P < 0.01. **(B)** Percentage of CD226^+^CD19^+^ B cells in pSS patients with positive and negative antibodies by flow cytometry(Student’s t-test and Mann-Whitney U test) *P < 0.05; **P < 0.01; ns: not significant (*P*≥0.05). **(C)** Percentage of CD226^+^CD19^+^ B cells in active and inactive pSS patients by flow cytometry (Mann-Whitney U test) **P < 0.01. **(D)** Percentage of CD226^+^CD19^+^ B cells in pSS patients before and after treat by flow cytometry (Student’s t-test). **(E)** The ROC curve of CD226^+^CD19^+^ B cells in discriminating pSS and HCs. **(F)** The ROC curve of CD226^+^CD19^+^ B cells in distinguishing disease activity of pSS patients.

Then, we analyzed the differences of CD226^+^ CD19^+^ B cells percentage in the peripheral blood of pSS patients with different clinical features and autoantibodies positive or negative. We found that the percentage of CD226^+^ CD19^+^ B cells in pSS patients with arthralgia, fatigue, decayed tooth, xerostomia, interstitial lung disease(ILD), leukopenia and high IgG was elevated compared to those without these clinical manifestations (**Figure 2A**). Among patients who were positive for anti-SSA/Ro52 antibody, anti-SSB/La antibody, and anti-centromere antibody, the percentage of CD226^+^ CD19^+^ B cells was significantly higher than that in patients who were negative for these antibodies (**Figure 2B**). The representative FACS plots of CD226^+^ CD19^+^ B cells percentage in the peripheral blood of pSS patients with different clinical features and auto-antibody statuses were displayed in **Supplementary Figure 2**. Moreover, compared to patients with inactive disease, those with active disease exhibited a marked increase in the percentage of CD226^+^ CD19^+^ B cells (**Figure 2C**). The ROC curve analysis revealed that CD226^+^ CD19^+^ B cells have significant discriminative capability in distinguishing pSS patients from HCs and in evaluating disease activity (**Figures 2E, F**). In addition, we followed up with seven patients who had received treatment for over two months and observed a downward trend in the percentage of CD226^+^ CD19^+^ B cells after treatment, however, there was no statistical significance (**Figure 2D**).

### 3.3 Abnormal distribution of CD226^+^ CD19^+^ B cell subsets in the peripheral blood of pSS patients

We detected the expression levels of CD226 in different B cell subsets in the peripheral blood of pSS patients and HCs by multi-color flow cytometry. B cell subsets were classified according to CD27/IgD and CD21/CD38 as follows: CD27^−^ IgD^+^ as naive B cells, CD27^+^ IgD^+^ as unswitched memory B cells, CD27^+^ IgD^−^ as switched memory B cells, CD27^−^ IgD^−^ as double-negative B cells and CD21^−^ CD38^+^ as plasmablasts (20, 21). The gating strategies of CD27, IgD, CD21 and CD38 on CD226^+^ CD19^+^ B cells are shown in **Supplementary Figure 3A**.

**Figure 3:**
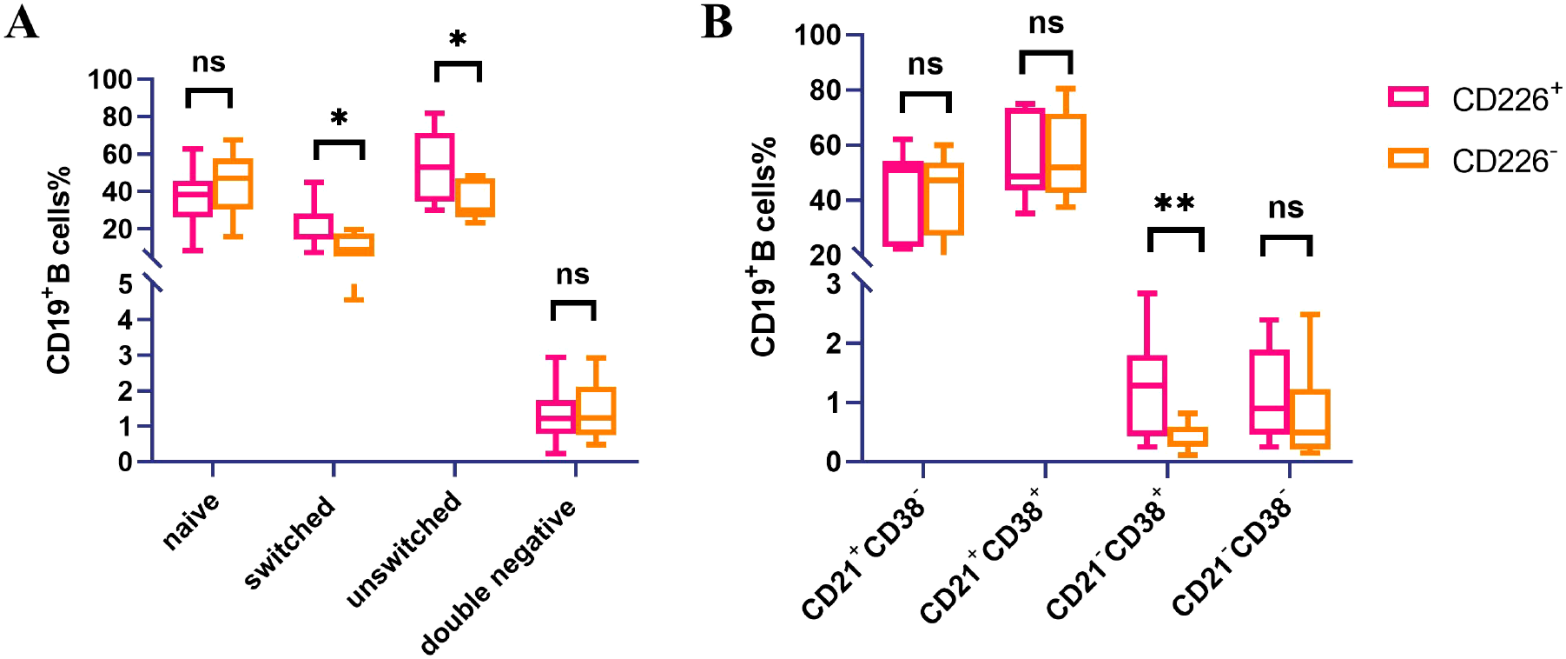
Distribution of CD226^+^ and CD226^−^CD19^+^ B cells subsets in peripheral blood of pSS patients (n=11). **(A)** Classification by CD27/IgD, the distribution of CD226^+^ and CD226^−^ B cells subsets in the peripheral blood of pSS patients (CD27^−^ IgD^+^ as naive B cells, CD27^+^ IgD^−^ as switched memory B cells,CD27^+^ IgD^+^ as unswitched memory B cells, and CD27^−^ IgD^−^ as double-negative B cells) (Student’s t-test) *P < 0.05; ns: not significant (*P*≥0.05). **(B)** Classification by CD21/CD38, the distribution of CD226^+^ and CD226^−^ B cells subsets in the peripheral blood of pSS patients(Student’s t-test) **P < 0.01; ns: not significant (*P*≥0.05).

The results showed that the percentage of CD226^+^ cells on switched memory B cells and unswitched memory B cells was higher than that of CD226^−^ cells in pSS patients(switched memory B cells%: 20.16% ± 12.38% *vs*. 10.53% ± 5.80%, *P*=0.03; unswitched memory B cells%: 52.63% ± 18.87% *vs*. 35.02% ± 10.11%, *P*=0.03)(**Figure 3A**). There was no significant difference in the expression of CD226 between pSS patients and HCs on naive B cells, unswitched memory B cells, switched memory B cells and double negative B cells (**Supplementary Table 1**). No significant difference was observed in the expression of CD226^+^ and CD226^−^ cells on naive B cells and double-negative B cells in the peripheral blood of pSS patients (**Supplementary Table 2**). Compared to CD226^−^cells, the percentage of CD226^+^ cells on plasmablasts was significantly elevated (1.30% ± 0.81% *vs*. 0.40% ± 0.22%,*P*=0.002, **Figure 3B**). In comparison with HCs, the percentage of CD226 on plasmablasts and CD21^−^ CD38^−^ B cells in pSS patients significantly increased (**Supplementary Table 1**). In addition, the distribution of CD226^+^ and CD226^−^ cells on B cell subsets did not exhibit a significant difference among HCs (**Supplementary Table 3**).

### 3.4 Association between CD226 expression on B cells and activated/inflammatory phenotypes in patients with pSS

In order to evaluate the association between CD226^+^ B cells expression and activated/inflammatory phenotypes in pSS patients, we measured the expression levels of costimulatory molecules(CD80,CD86 and CD137), activation markers(CD69), proliferation markers(Ki67), immunoglobulins (IgG and IgA), and proinflammatory cytokines (TNF-α and IFN-γ) in the peripheral blood of patients with pSS by fow cytometry.

**Figure 4:**
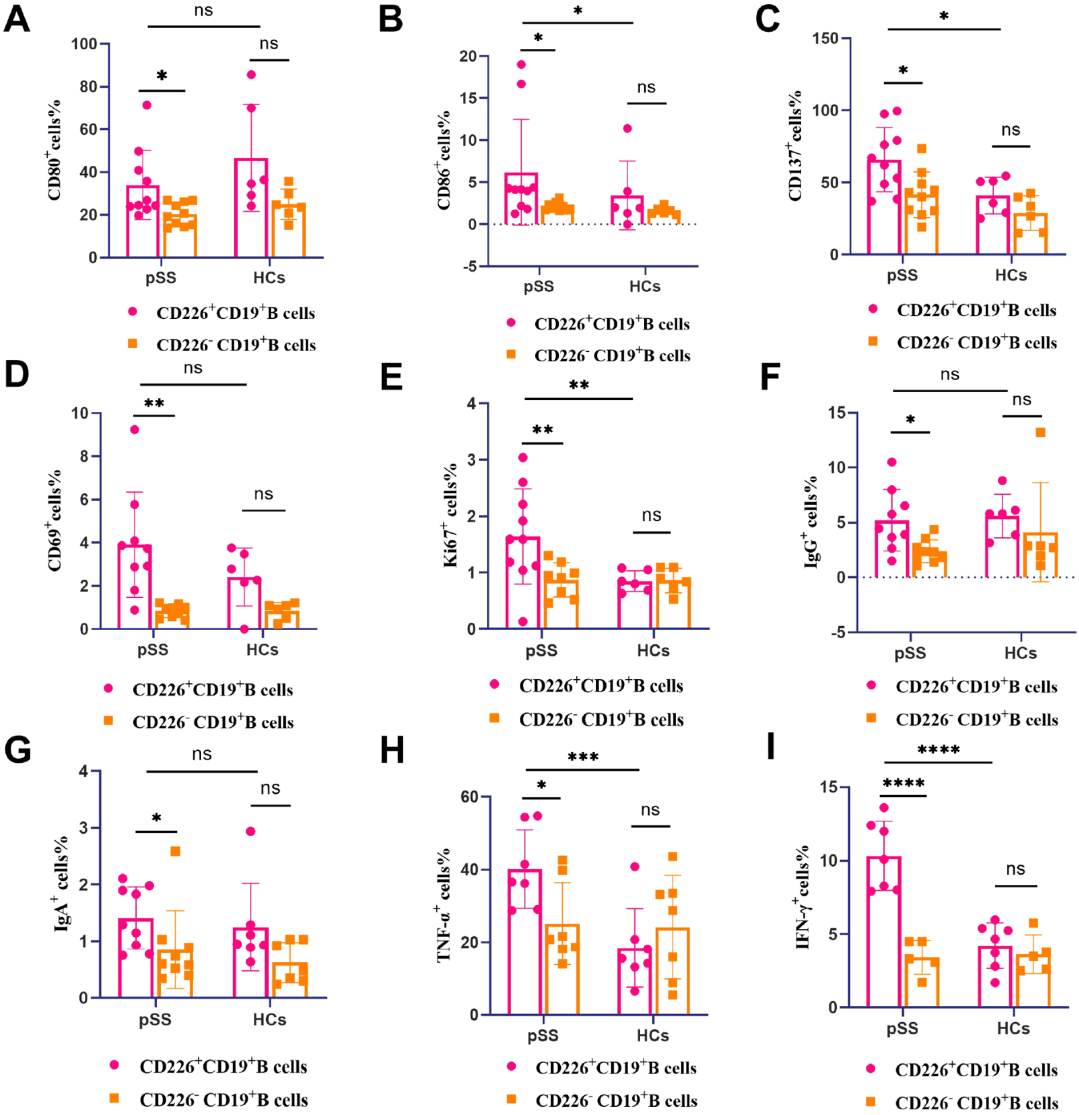
Association between CD226^+^ B cells expression and activated/inflammatory phenotypes in pSS patients. **(A-I)** Percentage of CD80 **(A)**, CD86 **(B)**, CD137 **(C)**, CD69 **(D)**, Ki67 **(E)**, IgG **(F)**, IgA **(G)**,TNF-α **(H)** and IFN-γ **(I)** on CD226^+^CD19^+^ B cells and CD226^−^CD19^+^ B cells in peripheral blood of pSS patients and HCs (Student’s t-test and Mann-Whitney U test) *P < 0.05; **P < 0.01; ***P<0.001; ****P<0.0001; ns: not significant (*P*≥0.05).

The gating strategies of CD80, CD86, CD137, CD69, Ki67, IgG, IgA, TNF-α and IFN-γ on CD226^+^ CD19^+^ B cells are presented in **Supplementary Figures 3B-E**. It was observed that in patients with pSS, CD226^+^ CD19^+^ B cells exhibited significantly elevated expression of costimulatory molecules(CD80, CD86, CD137), activation markers(CD69), proliferation markers(Ki67), immunoglobulin production (IgG, IgA), and pro-inflammatory cytokines (TNF-α, IFN-γ) compared to their CD226^−^ CD19^+^ B cell counterparts in the peripheral blood (**Figure 4**). No significant difference was detected in the expression levels of above indicators between CD226^+^ CD19^+^ B cells and CD226^−^ CD19^+^ B cells subsets in HCs (**Figure 4**). In comparison with HCs, the percentage of costimulatory molecules(CD86, CD137), proliferation markers(Ki67), and pro-inflammatory cytokines (TNF-α, IFN-γ) on CD226^+^ CD19^+^ B cells was significantly upregulated in the peripheral blood of pSS patients (**Figure 4**). The representative FACS plots of costimulatory molecules(CD80, CD86, CD137), activation markers(CD69), proliferation markers(Ki67), immunoglobulins (IgG, IgA), and pro-inflammatory cytokines (TNF-α, IFN-γ) of CD226^+^ CD19^+^ B cells and CD226^−^ CD19^+^ B cells in the peripheral blood of pSS patients and HCs were exhibited in **Supplementary Figure 4**. The representative FACS plots of costimulatory molecules (CD86, CD137), proliferation markers(Ki67), and pro-inflammatory cytokines (TNF-α, IFN-γ) of CD226^+^ CD19^+^ B cells in the peripheral blood of pSS patients and HCs are shown in **Supplementary Figure 5**.

### 3.5 The potential function of CD226^+^ CD19^+^ B cells in pSS

**Figure 5:**
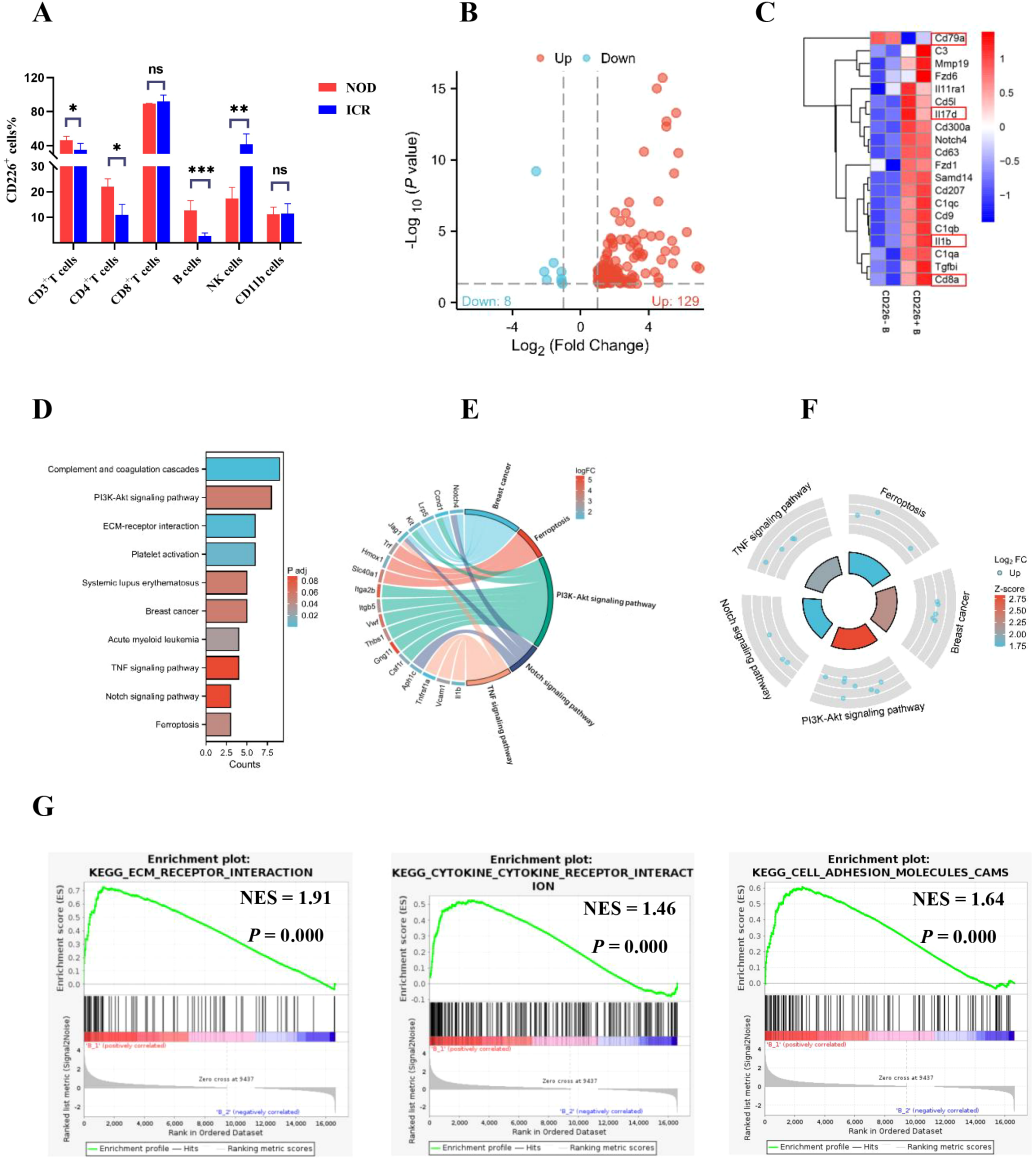
The microarray analysis for CD226^+^CD19^+^ and CD226^−^CD19^+^ B cell subsets. **(A)** Percentage of CD226 on different immunocytes in NOD (n=5) and ICR mice (n=5) by flow cytometry (Student’s t-test) *P < 0.05; **P < 0.01; ns: not significant (*P*≥0.05). **(B)** Volcano plot depicted differentially expressed genes (DEGs) in CD226^+^CD19**^+^** B cells and CD226^−^CD19**^+^** B cells sorted from splenocytes of NOD mice. Upregulated genes were labeled in red, and downregulated genes were labeled in blue. **(C)** Heatmap showed DEGs in CD226^+^CD19**^+^** B cells and CD226^−^CD19**^+^** B cells sorted from splenocytes of NOD mice. (red for high expression, blue for low expression). **(D–F)** KEGG enrichment analysis for CD226^+^CD19^+^ B cells sorted from splenocytes of NOD mice. **(G)** GSEA for the potential function of DEGs of CD226^+^CD19^+^ B cells sorted from splenocytes of NOD mice.

To investigate the functional role of CD226^+^ CD19^+^ B cells in pSS, CD226^+^ CD19^+^ and CD226^−^ CD19^+^ B cell subsets were isolated from splenic mononuclear cells of NOD mice via FACS, followed by transcriptome-wide microarray profiling to delineate their differential molecular signatures. Using multi-color flow cytometry, we first detected CD226 expression levels on major immunocyte subsets, including CD3^+^ T cells (CD4^+^ and CD8^+^ subsets), B220^+^ B cells, NK1.1^+^ NK cells, and CD11b^+^ monocytes in splenic mononuclear cells of NOD mice and ICR controls. In NOD mice, the percentage of CD226 on CD3^+^ T cells, CD4^+^ T cells and B220^+^ B cells was significantly higher than that of ICR mice, whereas the percentage of CD226 on NK1.1^+^ NK cells was markedly reduced in NOD mice relative to ICR mice(CD3^+^ T cells%:46.30% ± 4.48% *vs*. 35.16% ± 7.46%, *P*=0.0210; CD4^+^ T cells%: 22.06% ± 3.03% *vs*. 11.02% ± 4.08%, *P*=0.0013; B220^+^ B cells%: 12.81% ± 3.76% *vs*. 2.74% ± 1.15%,*P*=0.0004; NK1.1^+^ NK cells%: 17.38% ± 4.45% *vs*. 41.54% ± 3.97%,*P*=0.0032, **Figure 5A**). The representative FACS plots of CD226 on CD3^+^ T cells CD4^+^ T cells, B220^+^ B cells, and NK1.1^+^ NK cells in splenic mononuclear cells of NOD and ICR mice are displayed in **Supplementary Figure 6**. Through microarray analysis, we found that there were 129 upregulated DEGs and 8 downregulated DEGs between CD226^+^ CD19^+^ B cells and CD226^−^ CD19^+^ B cells (**Figure 5B**). The differential expression of Cd79a, Il17d, Il1b, and Cd8a between CD226^+^ CD19^+^ B cells and CD226^−^ CD19^+^ B cells held marked biological significance, potentially reflecting their distinct functional roles in immune regulation or disease pathogenesis (**Figure 5C**). KEGG pathway analysis demonstrated that CD226^+^ CD19^+^ B cells were significantly enriched in multiple critical biological pathways, including but not limited to complement and coagulation cascades, PI3K-Akt signaling pathway, extracellular matrix (ECM)-receptor interactions, and platelet activation mechanisms. Furthermore, these cells exhibited obvious associations with neoplastic pathologies (notably breast cancer and acute myeloid leukemia),as well as autoimmune disorders such as systemic lupus erythematosus (SLE)(**Figures 5D-F**). Gene set enrichment analysis (GSEA) revealed that CD226^+^ CD19^+^ B cells mediated their biological functions through significantly enriched signaling pathways, including ECM-receptor interaction, cytokine-cytokine receptor interaction, and cell adhesion molecules (CAMs), with false discovery rate (FDR)-adjusted p-values <0.05(**Figure 5G**).

## 4 Discussion

Extensive evidence underscored the pivotal role of B cells in coordinating multifaceted immunopathological mechanisms underlying pSS (22). The activation and effector functions of B cells are stringently regulated by costimulatory molecules, which comprise both activating and inhibitory receptors. These receptors modulate critical B cell processes, including sustaining tolerance, triggering activation, presenting antigens, assisting T cell functions, facilitating class-switch recombination, producing antibodies, and secreting cytokines, thereby orchestrating adaptive immune responses while preventing autoimmunity (23). It has been demonstrated that costimulatory molecules can facilitate B cell to participate in the pathogenesis and progress of various autoimmune diseases, and targeting regulation of costimulatory molecule signaling pathway may inhibit the function of B cells, which is expected to be a promising therapeutic approach for the treatment of autoimmune diseases (24). Liu et al. indicated that the CD40-CD40L signaling pathway enhances the activation of B cells and the production of IgG in SLE patients (25). Another research reported that CD19^+^ ICOSL^+^ B cells were implicated as essential players in the pathogenic process of rheumatoid arthritis, and inhibition of this signaling may suppress the pro-inflammatory response and ameliorate the course of arthritis (26). This study primarily evaluated and analyzed the correlation between CD226^+^ B cells and clinical characteristics as well as disease activity in patients with pSS. We further explored the immune phenotypic profiles and co-expression patterns of CD226^+^ B cells, aiming to identify potential therapeutic targets and biomarkers for pSS.

Our study revealed that patients with pSS exhibited significantly elevated expression levels of CD226^+^ CD14^+^ monocytes in peripheral blood compared to HCs, as previously detailed in our prior research. In addition, consistent with findings reported by Deng et al. (27), we also observed higher CD226 expression on T cell subsets in pSS patients compared to HCs. Intriguingly, in comparison with HCs, we further identified that CD226 expression was markedly increased on CD19^+^ B cells but significantly reduced on CD56^+^ NK cells in pSS patients. Our findings on mouse splenic immune cells were consistent with these results, except for CD8^+^ T cells and monocytes (**Figure 5A**). Furthermore, we further found that the expression level of CD226^+^ CD19^+^ B cells correlated more closely with the clinical characteristics, disease activity and prognosis of pSS patients. Lv et al. (28) have shown that CD40-CD40L costimulatory axis coordinated the class-switch and differentiation of B cells through type 3 innate lymphocytes and potentiated the biological function of B cells, which is similar to our research results. It was reported by Li et al. (29), a substantial number of CD226^+^ cells were identified in the muscle fiber tissue of patients with idiopathic inflammatory myopathy (IIM), and this was significantly correlated with the severity of muscle inflammation, which suggested that the costimulatory molecule CD226 was implicated in the pathogenesis of IIM. These studies have revealed that CD226 is dysregulated on immune cells of peripheral blood and infiltrating tissue across autoimmune diseases, which contributes to disease progression in autoimmune disorders.

CD226 is a costimulatory molecule mainly expressed on T cells and NK cells, and is also detected on B lymphocyte subsets (11). Despite increasing evidence implicated that CD226 participated in the pathogenesis of autoimmune diseases via T cells or NK cells (27, 30–35), there remained a paucity of studies of the correlation between CD226^+^ B lymphocytes and autoimmune disease. Nakano et al. (36) demonstrated that elevated proportions of CD226^+^ B lymphocytes were significantly associated with enhanced disease activity and unfavorable clinical prognosis in patients with SLE. Building upon existing evidences and our preliminary findings (14, 15), this study delved into the immunophenotypic profile and functional implications of CD226 on B lymphocytes in pSS. Our study showed that CD226^+^ cells exhibited a significantly higher proportional distribution compared to their CD226^−^cells within switched memory B cells, unswitched memory B cells, and plasmablasts in patients with pSS (**Figure 3**). Naive B cells demonstrated a limited capacity to produce immunoglobulins whereas upon stimulation, unswitched memory B cells generated substantial IgM and switched memory B cells secreted a large number of IgG, with both exhibiting unique potential to differentiate into plasmablasts (37, 38). Consistent with our findings, patients with SLE exhibited elevated proportion of CD226^+^ B cells in both switched memory B cells and plasmablasts, indicating a pathogenic role of CD226 on B cell subsets in SLE-driven autoimmunity (36). Previous *in vitro* study has demonstrated that CD226 expression on primary B cells and plasmablasts is significantly upregulated following Epstein-Barr virus (EBV) infection, and this observation suggested that CD226 played a critical role in modulating activation and differentiation of B cell during EBV infection (39). EBV infection is closely associated with the pathogenesis of pSS. It is reported that active EBV infection is selectively correlated with ectopic lymphoid structures in the salivary glands of pSS patients, promoting the local survival and differentiation of disease-specific autoreactive B cells (40). Furthermore, persistent EBV infection elevates the risk of lymphoma development in pSS patients (41). Accumulating evidence indicated that external triggers, including viral infections and environmental factors drove the differentiation of naïve B cells into functionally distinct subsets, particularly antibody-secreting plasmablasts and long-lived plasma cells, and this activation cascade culminated in the generation of pathogenic autoantibodies and tissue-deposited immune complexes, which subsequently initiated inflammation and autoimmune tissue damage (42). Our findings demonstrated that CD226 critically drove the differentiation of B cells into antibody-secreting plasmablasts in patients with pSS, thereby amplifying the production of pathogenic autoantibodies and facilitating antibody class switching.

The T cell-dependent activation of B cells requires costimulatory signals. These signals are mediated by the interaction of costimulatory receptors (such as CD28 and CTLA-4 on T cells) and ligands (such as CD80 and CD86 on B cells), which provide the critical second signal required for B cell activation. The interaction between CD80/CD86 and costimulatory receptors on T cells is integral for the formation of the immunological synapse. Furthermore, upregulation of either CD80 or CD86 enhances the capacity of B cell antigen-presenting, thereby strengthening T-B cell interactions and promoting the differentiation, survival, and proliferation of both B cells and T cells (43). CD137 (4-1BB), a member of TNFSF, functions as a costimulatory molecule that activated B cell survival, proliferation, and cytokine secretion through ligand binding (CD137L/4-1BBL)-dependent signaling (44, 45). *In vitro* studies utilizing the experimental autoimmune encephalomyelitis model demonstrated that CD137 signaling significantly augmented B cell activation, proliferation, and pro-inflammatory cytokine secretion, suggesting its critical role in amplifying B cell-mediated immunopathology in neuroinflammatory conditions (46) CD69 is a membrane-bound type II C-lectin receptor and serves as a classical early marker of lymphocyte activation (47). Ki67, a nuclear protein antigen expressed throughout the cell cycle in proliferating cells, is a well-established marker of cellular proliferation (48, 49). CD226^+^ B cells from pSS patients exhibited significantly higher co-expression of CD86, CD137, and Ki67 compared to HCs. This coordinated upregulation of costimulatory molecules and proliferation markers suggests a potential association between CD226 expression and an activated B cell phenotype in pSS. The observed molecular signature, characterized by enhanced costimulation (CD86/CD137) and proliferative capacity (Ki67), may contribute to sustained immune activation in pSS pathogenesis. However, further functional studies are required to determine whether CD226 plays a direct role in regulating these B cell functions in pSS.

Furthermore, we observed that CD226^+^ CD19^+^ B cells from pSS patients exhibited higher proportions of IgG and IgA compared to CD226^−^CD19^+^ B cells, implicating that CD226 may be involved in Ig class switching in B cells of pSS patients. Upon antigen stimulation, activated IgG^+^ B cells undergo rapid proliferation and differentiation into plasmocytes, generating high-titer, high-affinity IgG antibodies, and research has demonstrated that mIgG1 ubiquitination plays a crucial role in promoting the survival and expansion of germinal center B cells (50, 51). Recent studies have demonstrated that CD226^+^ T cells in the peripheral blood of patients with primary biliary cholangitis (PBC) exhibit enhanced pro-inflammatory activity and proliferative capacity compared to their CD226^−^ counterparts. Furthermore, blockade of CD226 signaling significantly attenuated both effector function and proliferation of T cells, underscoring its critical role in T cell-mediated immune responses in PBC (52). In patients with primary antiphospholipid syndrome, CD226 expression was significantly upregulated on CD56^bright^ NK and NK-like cells. Moreover, the CD226^+^ CD56^bright^ NK cell subset exhibited higher expression levels of CD69 and CD25, as well as enhanced IFN-γ production and CD107a degranulation capacity compared to their CD226^−^ counterparts (30). In patients with tuberculosis (TB) infection, peripheral blood T cells and NK cells exhibited significantly elevated expression of CD226 compared to HCs. The CD226^+^ cell subsets demonstrated markedly enhanced IFN-γ production and CD107a degranulation capacity relative to their CD226^−^ counterparts. These findings suggested that CD226 may serve as a potential predictive biomarker for disease progression and clinical outcomes in TB, likely through mediating the cytotoxic functions of both T cells and NK cells (53). The latest research has shown that CD226 blockade enhances the function of regulatory T cells, reduces the cytotoxicity of effector T cells, and lower the incidence of spontaneous diabetes in NOD mouse models (54). The findings in our study demonstrated that CD226^+^ CD19^+^ B cells in pSS patients exhibited significantly higher pro-inflammatory cytokine production capacity compared to CD226^−^ CD19^+^ B cells. The elevated inflammatory potential of CD226^+^ B cells, as evidenced by their enhanced cytokine secretion profile, implicated this subset in perpetuating the pro-inflammatory microenvironment characteristic of pSS. The GSEA also revealed that CD226^+^ CD19^+^ B cells mediated their biological functions through cytokine-cytokine receptor interaction (**Figure 5G**).

Notwithstanding these findings, certain limitations of the this study should be acknowledged: First, our study lacks mechanistic insights into how CD226 precisely regulates the biological function of B cell. Further investigations are required to delineate the underlying molecular pathways, which may reveal more suitable immunomodulatory strategies and precise therapeutic targets for alleviating pSS. Second, our study demonstrates that CD226 is significantly upregulated on B cells from both peripheral blood and salivary glands in pSS patients. Moreover, its expression level positively correlates with disease activity and severity. These findings suggest that CD226 may contribute to pSS pathogenesis by enhancing B cell effector functions. However, although we confirmed aberrant CD226 overexpression on splenic B cells in the mouse model, functional intervention studies are still lacking to further characterize the biological properties of CD226^+^ B cells. Finally, the study’s generalizability may be limited by relatively small sample sizes of both human subjects and animal models, which may introduce potential bias to some findings. Importantly, our findings only apply to treatment-naïve patients at initial diagnosis, as we strictly excluded those receiving any immunomodulatory therapies. Consequently, the observed immunological profiles may not be generalizable to treated populations or later disease stages. Notably, we observed aberrant CD226 expression not only in B cells but also in T cells and NK cells from pSS patients and NOD mice. Future studies should expand cohort sizes to validate these observations, and investigate how CD226-mediated dysregulation in multiple immune cell subsets collectively contributes to pSS development.

## 5 Conclusion

In summary, a novel B cell subset, CD226^+^ CD19^+^ B cells is identified in our study. This subset is closely associated with clinical features, disease activity, and prognosis in pSS patients. These cells exhibit heightened activation and pro-inflammatory phenotypes. Our findings highlight CD226^+^ B cells as a potential therapeutic target and biomarker for pSS, offering new avenues for disease-modifying interventions.

## Data Availability

The data presented in this study are deposited in the GEO repository, accession number GSE303018.
