# Supplementary material for: CD226+ B cells in primary Sjögren’s syndrome: a key player in clinical manifestations and disease pathogenesis
Source: Front Immunol. 2025 Jul 25;16:1623774. doi: 10.3389/fimmu.2025.1623774 (PMC12331654; doi:10.3389/fimmu.2025.1623774)
Supplement: Supplementary file 1 [file DataSheet1.docx]

**Supplementary materials**


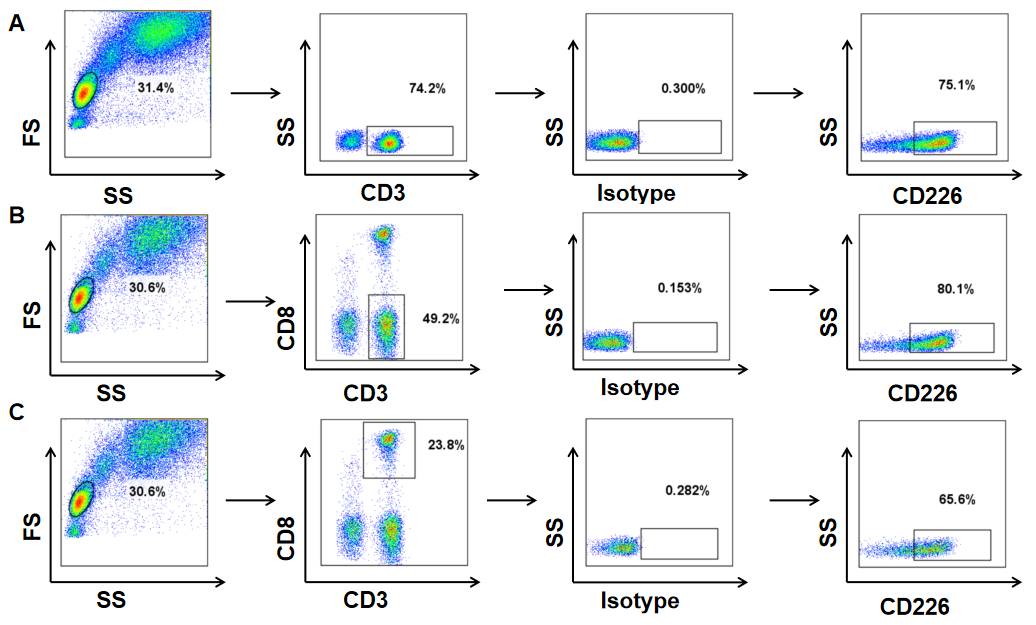


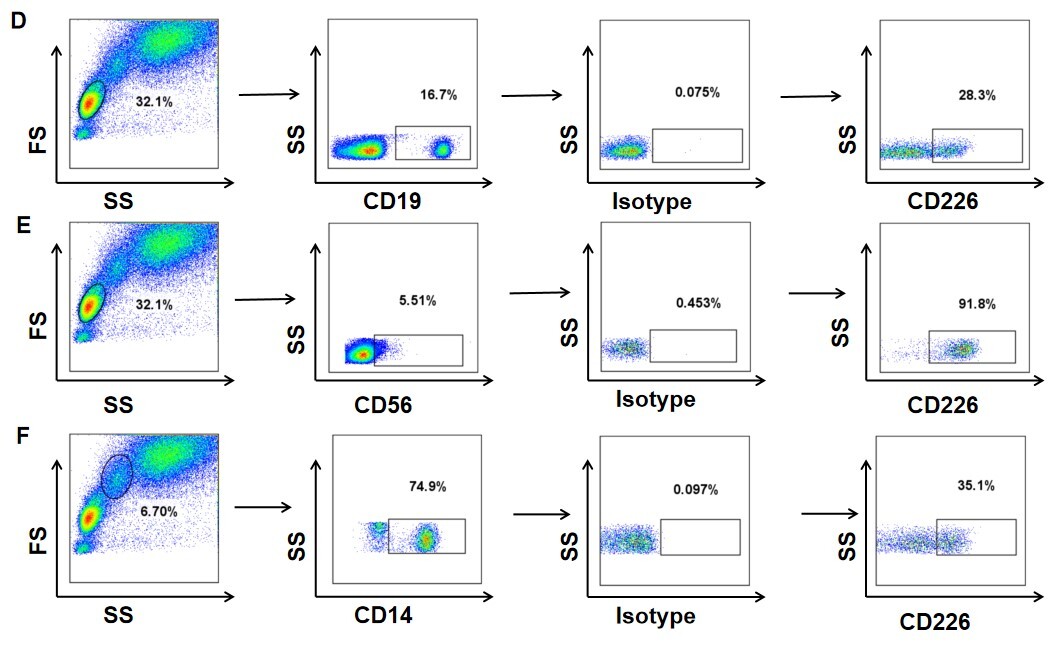


**Supplementary Fig.1 The gating strategies for CD226 on CD3^+^T cells(A), CD3^+^CD8^-^T cells(B), CD8^+^T cells(C), CD19^+^B cells(D), CD56^+^NK cells(E) and CD14^+^ monocytes(F).**

**Table 1 Comparison of distribution of CD226^+^B cell subsets in the PB between pSS and HCs**

|  | pSS | HCs | t | *P* |
| --- | --- | --- | --- | --- |
| CD27/IgD |  |  |  |  |
| navie B cells (%) | 39.38±15.03 | 30.29±13.55 | 1.21 | 0.25 |
| memory B cells (%) | 72.23±16.28 | 61.78±11.98 | 1.31 | 0.21 |
| switched (%) | 36.59±11.51 | 48.87±17.61 | 1.64 | 0.12 |
| unswitched (%) | 21.44±12.26 | 25.35±13.37 | 0.59 | 0.56 |
| double-negative B cells (%) | 1.43±0.75 | 1.05±1.04 | 0.85 | 0.41 |
| CD21/CD38 |  |  |  |  |
| CD21^+^B cells (%) | 97.54±1.42 | 99.10±0.61 | 2.54 | 0.02* |
| CD38^+^B cells (%) | 55.47±17.32 | 53.13±10.13 | 0.30 | 0.77 |
| CD21^+^CD38^－^B cells (%) | 42.28±15.80 | 38.82±10.49 | 0.48 | 0.64 |
| CD21^+^CD38^+^B cells (%) | 59.56±13.50 | 49.02±10.76 | 1.49 | 0.16 |
| CD21^－^CD38^+^B cells (%) | 1.41±0.77 | 0.48±0.25 | 2.58 | 0.02* |
| CD21^－^CD38^－^B cells (%) | 1.12±0.73 | 0.44±0.29 | 2.15 | 0.04* |

**Table 2 Comparison of distribution of CD226^+^ B cells and CD226**^－^**B cells subsets in the PB of pSS patients**

|  | CD226^+^  CD19^+^ B cells | CD226^－^  CD19^+^ B cells | t | *P* |
| --- | --- | --- | --- | --- |
| CD27/IgD |  |  |  |  |
| navie B cells (%) | 36.55±17.07 | 44.12±16.73 | 1.05 | 0.31 |
| memory B cells (%) | 68.00±19.82 | 60.22±21.44 | 0.80 | 0.44 |
| switched (%) | 52.63±18.87 | 35.02±10.11 | 2.47 | 0.03* |
| unswitched (%) | 20.16±12.38 | 10.53±5.80 | 2.34 | 0.03* |
| double-negative B cells (%) | 1.32±0.79 | 1.41±0.74 | 0.28 | 0.78 |
| CD21/CD38 |  |  |  |  |
| CD21^+^B cells (%) | 97.54±1.42 | 98.95±0.71 | 2.95 | 0.02* |
| CD38^+^B cells (%) | 55.47±17.32 | 57.23±16.91 | 0.24 | 0.81 |
| CD21^+^CD38^－^B cells (%) | 42.28±15.80 | 41.04±14.68 | 0.19 | 0.85 |
|  | CD226^+^  CD19^+^B cells | CD226^－^  CD19^+^B cells | t | *P* |
| CD21^+^CD38^+^B cells (%) | 55.38±15.24 | 56.83±15.84 | 0.22 | 0.83 |
| CD21^－^CD38^+^B cells (%) | 1.30±0.81 | 0.40±0.22 | 3.59 | 0.002** |
| CD21^－^CD38^－^B cells (%) | 1.12±0.73 | 0.77±0.71 | 1.14 | 0.27 |

**Table 3 Comparison of distribution of CD226^+^ B cells and CD226**^－^**B cells subsets in the PB of HCs**

|  | CD226^+^  CD19^+^ B cells | CD226^－^  CD19^+^ B | t | *P* |
| --- | --- | --- | --- | --- |
| CD27/IgD |  |  |  |  |
| navie B cells (%) | 26.01±16.76 | 31.84±19.02 | 0.61 | 0.55 |
| memory B cells (%) | 66.20±15.84 | 57.80±14.99 | 1.02 | 0.33 |
| switched (%) | 48.87±17.61 | 45.32±15.66 | 0.37 | 0.72 |
| unswitched (%) | 23.09±13.54 | 19.74±11.56 | 0.50 | 0.63 |
| double-negative B cells (%) | 1.05±1.04 | 1.41±1.34 | 0.53 | 0.61 |
| CD21/CD38 |  |  |  |  |
| CD21^+^B cells (%) | 99.10±0.61 | 99.28±0.59 | 0.53 | 0.61 |
| CD38^+^B cells (%) | 53.13±10.13 | 50.80±10.02 | 0.40 | 0.70 |
| CD21^+^CD38^－^B cells (%) | 42.90±14.44 | 43.74±14.02 | 0.11 | 0.91 |
| CD21^+^CD38^+^B cells (%) | 52.33±12.59 | 51.07±9.98 | 0.19 | 0.85 |
| CD21^－^CD38^+^B cells (%) | 0.98±0.87 | 0.35±0.28 | 1.96 | 0.07 |
| CD21^－^CD38^－^B cells (%) | 0.44±0.29 | 0.92±0.75 | 0.79 | 0.45 |

**A**


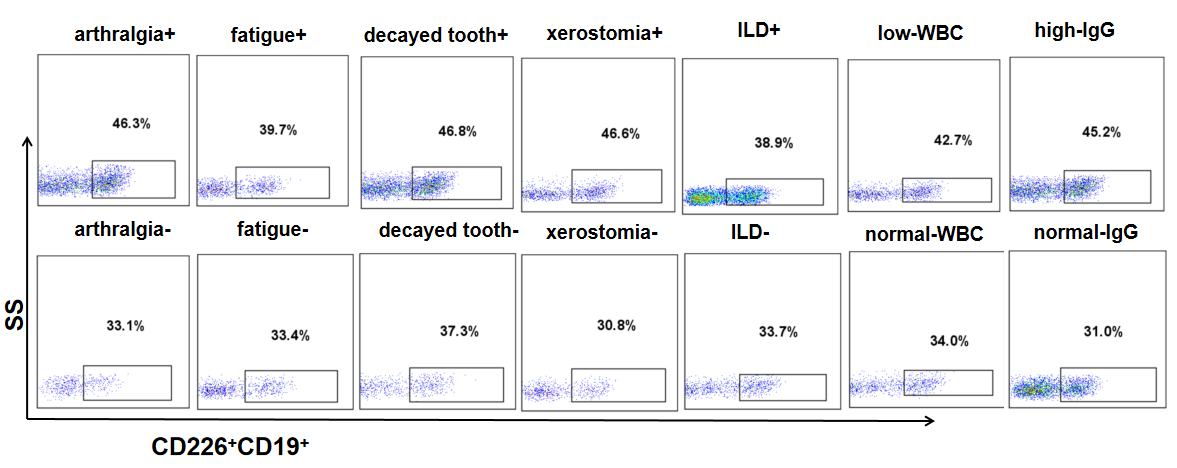


**B**


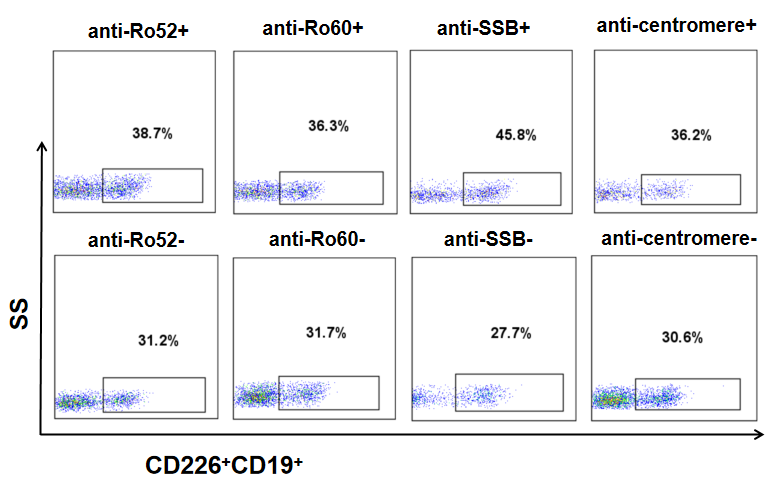


**Supplementary Fig.2:** The representative FACS plots of CD226^+^ CD19^+^ B cells percentage in the PB of pSS patients with different clinical features (A) and positive and negative auto-antibodies(B).

**A**


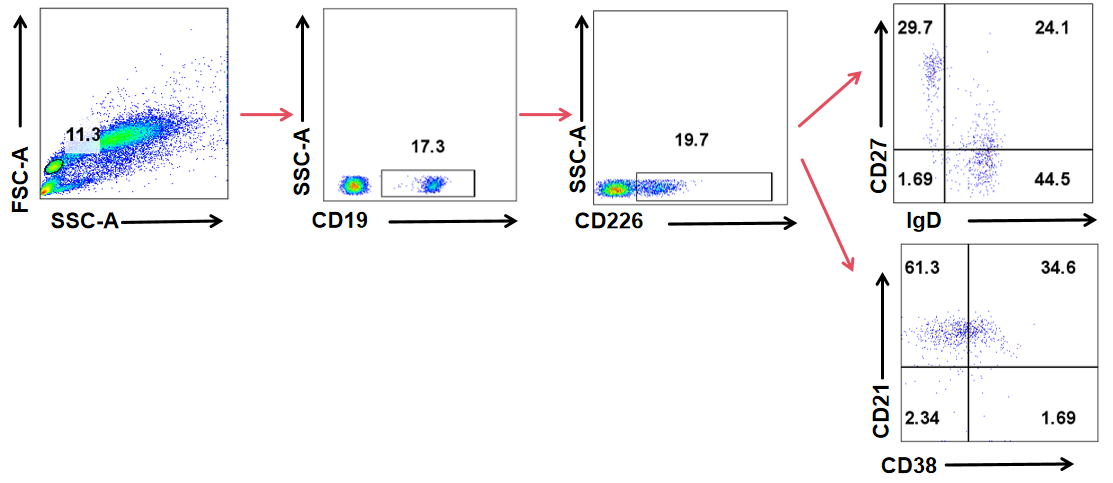


**B**


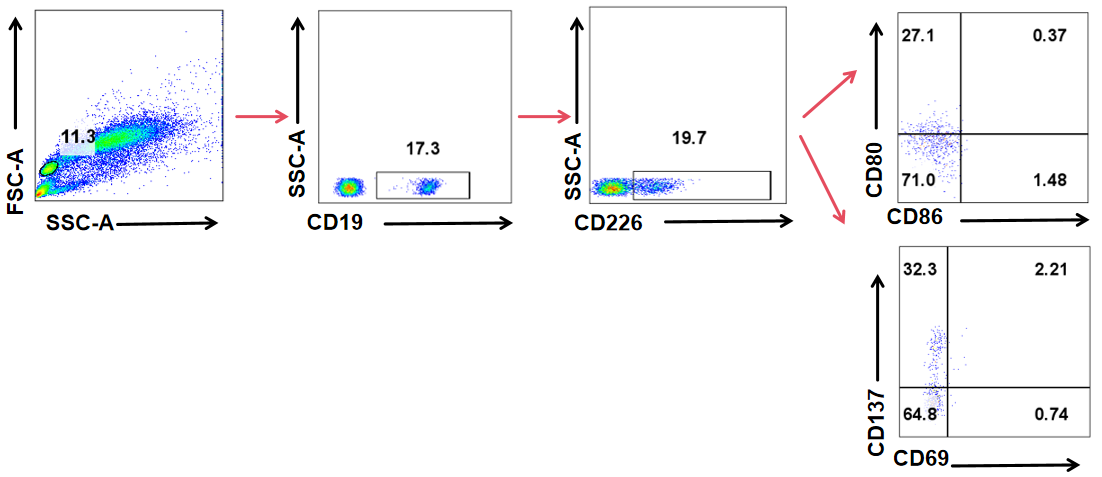


**C**


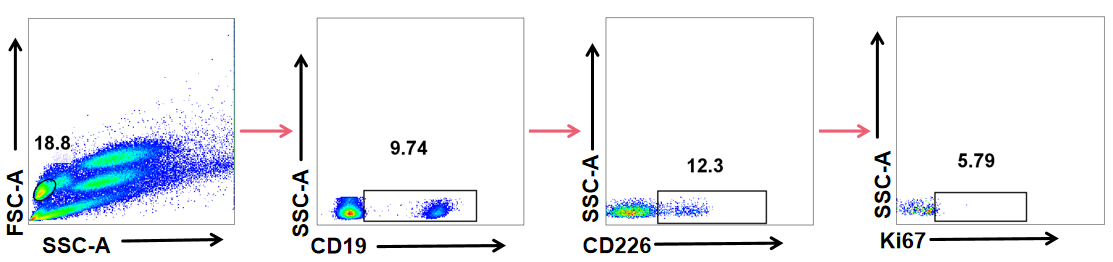


**D**


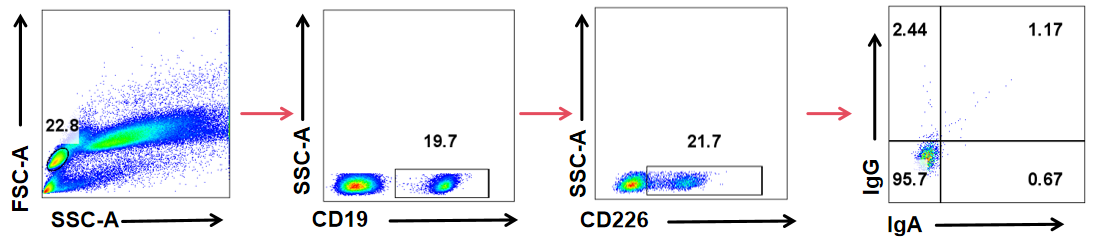


**E**


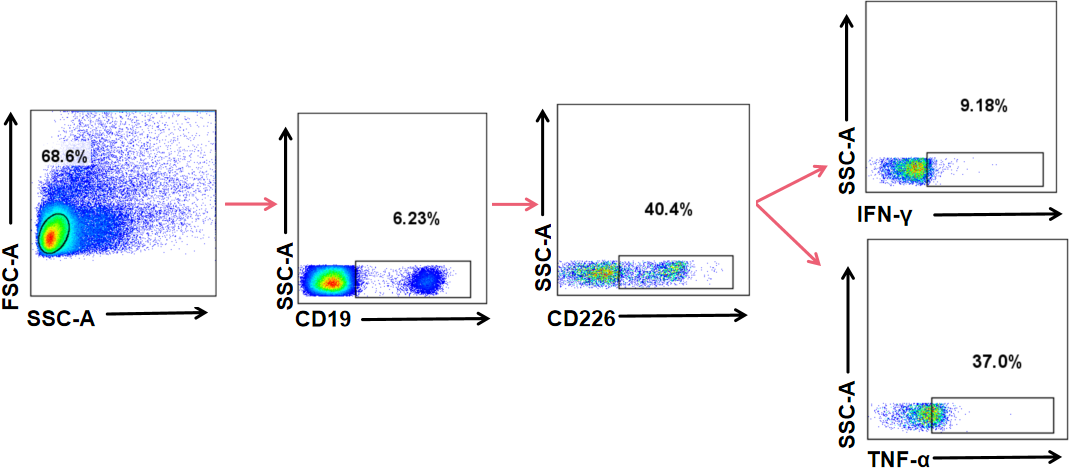


**Supplementary Fig.3 :** The gating strategies of CD27,IgD,CD21,CD38(A),CD80,

CD86,CD137,CD69(B),Ki67(C),IgG,IgA(D) and IFN-γ,TNF-α(E) on CD226^+^

CD19^+^B cells.

**A**


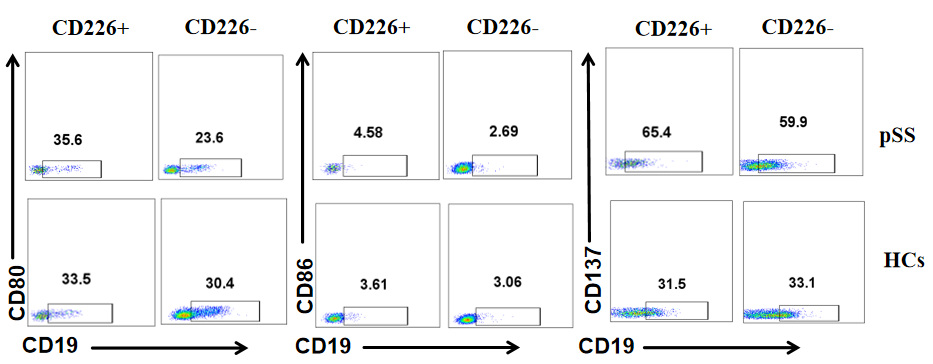


**B**


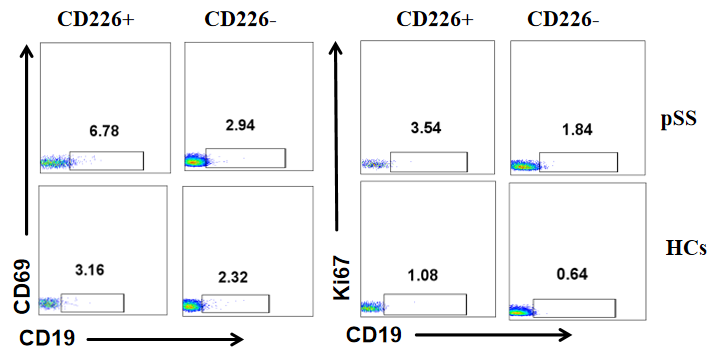


**C**


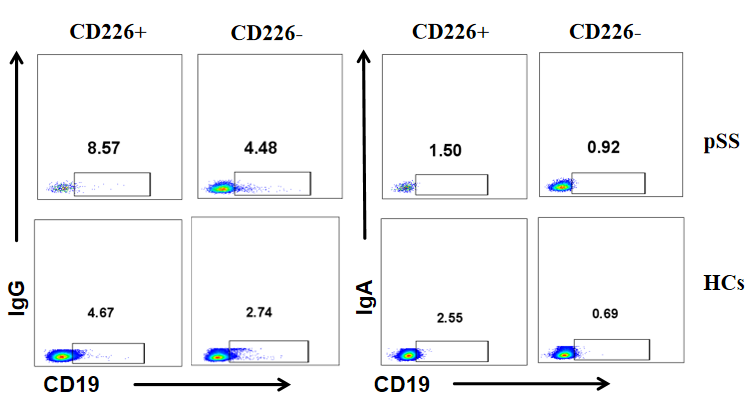


**D**


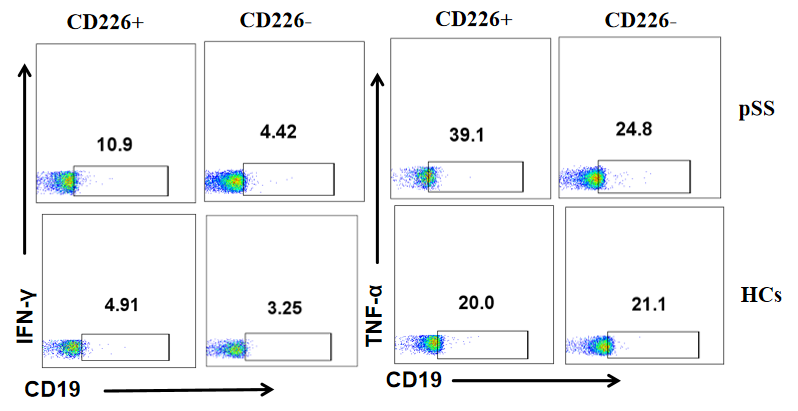


**Supplementary Fig.4 :** The representative FACS plots of costimulatory molecules (A),activation and proliferation markers(B), IgG and IgA(C) and pro-inflammatory cytokines(D) of CD226^+^CD19^+^ B cells and CD226^−^CD19^+^ B cells in the PB of pSS patients and HCs.

**A B**


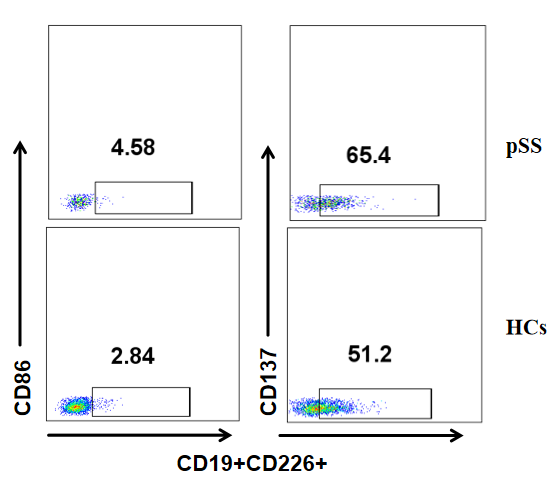

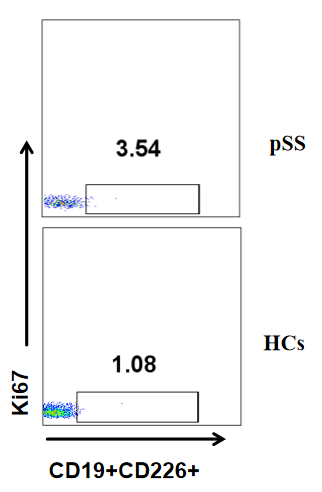


**C**


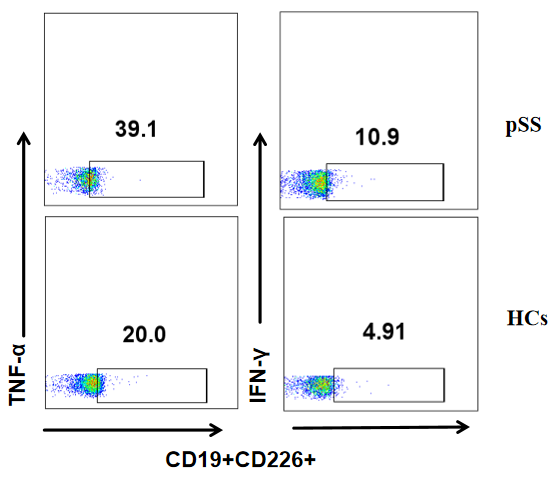


**Supplementary Fig.5 :** The representative FACS plots of costimulatory molecules (A),proliferation markers(B), and pro-inflammatory cytokines (C) of CD226^+^ CD19^+^ B cells in the PB of pSS patients and HCs.


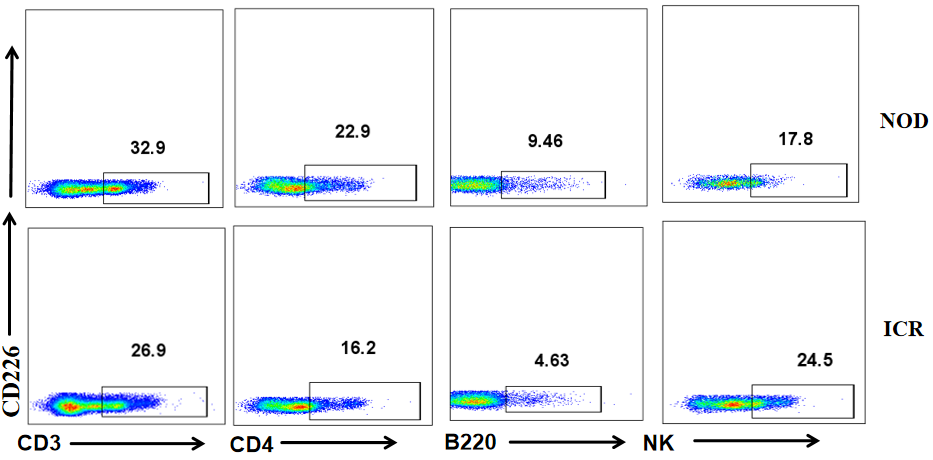


**Supplementary Fig.6:** The representative FACS plots of CD226 on CD3^+^ T cells CD4^+^ T cells , B220^+^ B cells, and NK1.1^+^ NK cells in splenic mononuclear cells of NOD mice and ICR controls.

**DAPI CD20 CD226 Merge**


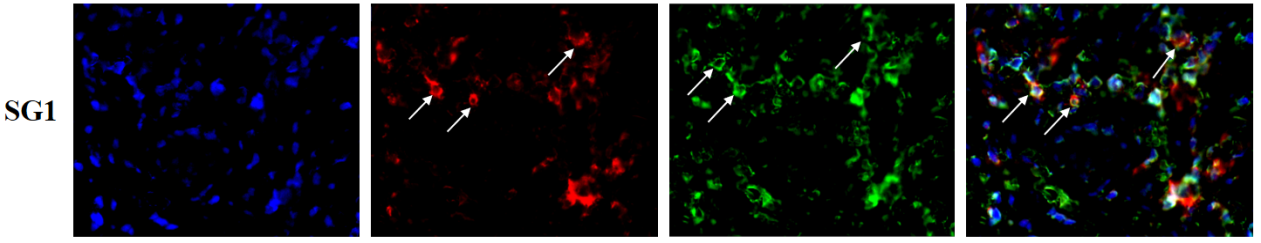


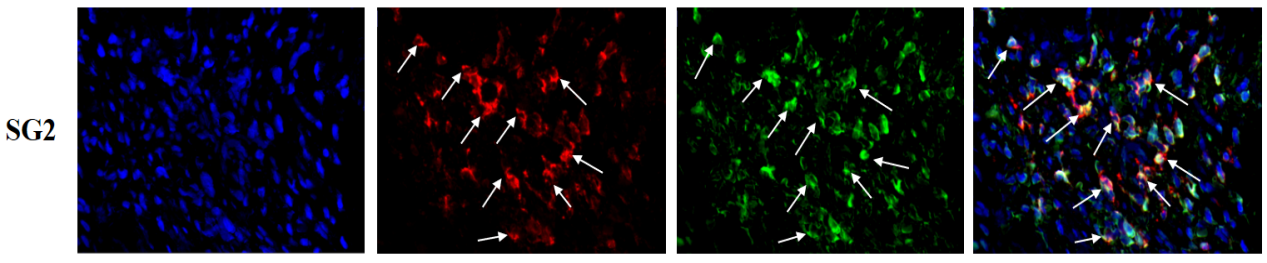


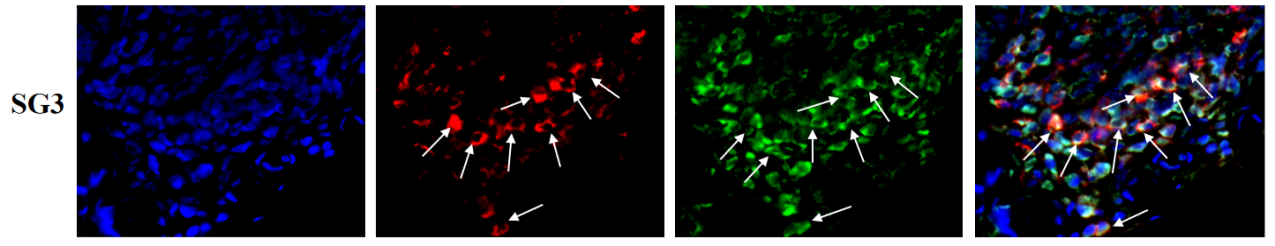


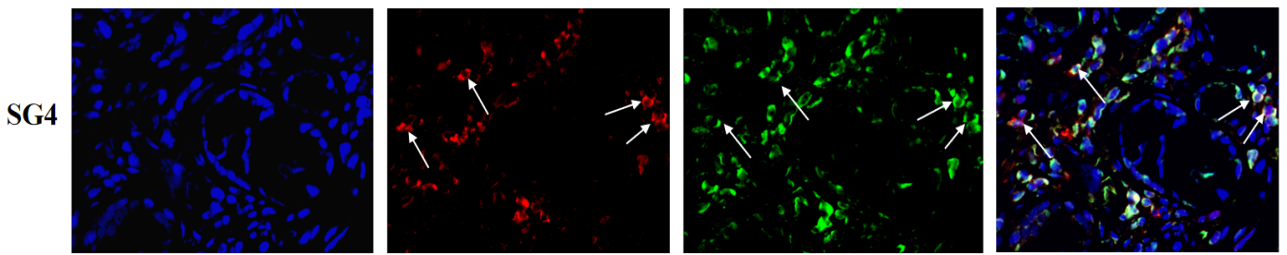


**Supplementary Fig.7:**The expression of CD226^+^CD20^+^ B cells in salivary gland of patients with pSS by immunoﬂuorescence staining (blue for DAPI, red for CD20, green for CD226).
